# Diagnóstico de COVID-19 ¿Qué hemos aprendido tras dos años de pandemia?

**DOI:** 10.1515/almed-2022-0048

**Published:** 2022-06-13

**Authors:** Melania Iñigo, Gabriel Reina, José Luís Del Pozo

**Affiliations:** Departamento de Microbiología, Clínica Universidad de Navarra, Madrid, España; Departamento de Microbiología, Clínica Universidad de Navarra. ISTUN, Instituto de Salud Tropical, Universidad de Navarra. IdiSNA, Instituto de Investigación Sanitaria de Navarra, Pamplona (Spain); Sección de Enfermedades Infecciosas, Departamento de Microbiología, Clínica Universidad de Navarra. IdiSNA, Instituto de Investigación Sanitaria de Navarra, Pamplona, España

El mundo se ha visto amenazado por una crisis sanitaria desde que, a finales de 2019, surgió y comenzó a extenderse el nuevo coronavirus 2019 (2019-nCoV) o coronavirus del síndrome respiratorio agudo grave 2 (SARS-CoV-2). A fecha del 10 de abril de 2022, se han documentado más de 496 millones de casos confirmados y más de 6 millones de muertes en las seis regiones de la OMS. Sin embargo, el impacto total de la pandemia es notablemente mayor de lo que indican las muertes registradas. Aunque entre el 1 de enero de 2020 y el 31 de diciembre de 2021 se reportaron 5,94 millones de muertes a nivel mundial por COVID-19, en un estudio reciente se estima que en realidad han fallecido 18,2 millones de personas en todo el mundo debido a la pandemia (medidas en términos de exceso de mortalidad durante dicho periodo) [1].

Las vacunas han reducido considerablemente las muertes relacionadas con COVID-19, habiéndose administrado más de 10.000 millones de dosis en todo el planeta. No obstante, la distribución de las vacunas ha sido notablemente desigual. La mayoría de los países desarrollados han vacunado e incluso administrado dosis de refuerzo a la mayor parte de sus poblaciones, mientras que los países más pobres presentan índices de vacunación extremadamente bajos. En los últimos meses se ha producido un punto de inflexión en la pandemia, debido a la elevada contagiosidad de las nuevas variantes, menos agresivas, sumado al hecho de que una gran proporción de la población está inmunizada gracias a las vacunas o debido a la infección natural. Por ello, numerosos gobiernos locales han relajado las restricciones sanitarias. Sin embargo, la experiencia y los datos disponibles indican que surgirán nuevas variantes, generando nuevos brotes, con efectos imprevisibles en las estrategias de tratamiento y prevención.

Desde que se documentaron los primeros casos, el diagnóstico rápido y preciso de infección por SARS-CoV-2 ha sido prioritario. Se han desarrollado diversos métodos, cada uno con sus fortalezas y limitaciones, dependiendo de la población diana, el escenario epidemiológico y los tiempos de respuesta. La reacción en cadena de la polimerasa en tiempo real (RT-qPCR) identifica la presencia de ARN de SARS-CoV-2 en muestras biológicas. Se considera la prueba diagnóstica de referencia debido a su elevada especifidad y sensibilidad. La RT-qPCR se basa en la detección específica de diferentes regiones del genoma del virus, principalmente de genes codificantes de proteínas estructurales, como la proteína S (*Spike*, espícula), proteína E (envoltura) y proteína N (nucleocápside), preferiblemente con la detección simultánea de varias dianas para evitar los falsos negativos. Aunque la RT-qPCR es la prueba de referencia, la necesidad de personal cualificado y de equipos especiales, el coste de la técnica y los prolongados tiempos de respuesta han obligado a desarrollar otras técnicas para poder atender a la elevada demanda de pruebas diagnósticas que se produce en una situación de pandemia [2].

Las pruebas de detección de antígenos (AgT) son inmunoensayos que localizan la presencia de un antígeno viral concreto. Estas pruebas se realizan de forma rápida y en el mismo lugar de asistencia, con tiempos de respuesta inferiores a los 30 minutos y bajo coste. Las AgT suelen ser menos sensibles que las técnicas moleculares por lo que se recomienda realizarlas durante los primeros días con síntomas (cuando la carga viral es mayor), con el fin de detectar a los individuos con mayor presencia viral (mayor riesgo de transmisión), y cuando se producen brotes en lugares cerrados. Mientras que las técnicas moleculares pueden ser excesivamente sensibles y generar resultados positivos durante mucho tiempo, detectando a individuos que ya no son contagiosos, las AgT reflejan con mayor precisión la capacidad de contagio de un paciente. Por ello, su uso también se recomienda en la última fase de la infección, para finalizar el aislamiento de los casos positivos. Además, son pruebas cualitativas que no permiten cuantificar la cantidad de antígenos [3, 4]. A diferencia de las pruebas AgT, las técnicas moleculares permiten una semicuantificación de la carga viral presente en la muestra, aplicando un valor umbral (Ct): valores elevados de Ct representan una baja carga de ARN viral y un menor riesgo de contagio (un valor de Ct>33–34 sugiere la desaparición de la capacidad de contagio). Esto puede corresponder al periodo de incubación, a la fase de convalecencia o a la replicación primaria del virus en otras regiones del cuerpo (vías respiratorias bajas). Por otro lado, unos valores de Ct bajos corresponden a una elevada carga viral, lo que supone una mayor capacidad de transmisión. Aunque existen pocos datos sobre la relación entre la carga viral de un paciente y su pronóstico, como la gravedad de la enfermedad o la tasa de mortalidad, algunos autores indican una relación inversamente proporcional entre el valor de Ct y la mortalidad. Cabe señalar que los valores de Ct se suelen ver afectados por variables preanalíticas (obtención de la muestra, condiciones de transporte, etc.), analíticas (carga de ARN viral en las muestras, diseño de los cebadores, reactivos de extracción y RT-qPCR empleados, etc.) y postanalíticas (principalmente, interpretación de los resultados). Por tanto, la estandarización de todas estas variables es esencial para una adecuada interpretación de los valores de Ct [5].

Por otro lado, continuamente aparecen nuevas variantes del virus debido a las numerosas mutaciones genéticas de la cepa original de Wuhan. El CDC y la OMS han clasificado algunas de estas nuevas cepas como variantes de preocupación (VOC, por sus siglas en inglés). Estas mutaciones pueden afectar a algunas de las características funcionales del virus, como la infectividad, inducir una mayor capacidad para evadir la respuesta de los anticuerpos neutralizantes generados por infección natural y/o por la vacuna, o incluso seleccionar variantes con mutaciones resistentes a los fármacos. Aunque la secuenciación masiva (NGS, por sus siglas en inglés) se considera la prueba de referencia para la identificación de variantes del SARS-CoV-2, se trata de una técnica compleja, solo disponible en los laboratorios más especializados. Para estudiar las nuevas variantes, se han desarrollado múltiples kits comerciales de RT-qPCR, lo que permite una tipificación más rápida y ampliada de las cepas circulantes. La NGS permite confirmar los resultados de la RT-qPCR y realizar un seguimiento de la aparición y dinámica de las nuevas variantes del SARS-CoV-2. Teniendo en cuenta la dinámica de las mutaciones virales y la rapidez con las que aparecen nuevas VOC, es necesario realizar una vigilancia continua de las nuevas variantes que vayan apareciendo [6].

Por otro lado, la detección de anticuerpos frente al SARS-CoV-2 permite identificar marcadores específicos de respuesta a la vacuna (anticuerpos contra la proteína *Spike* –Anti-S-), dado que la mayoría de las vacunas se basan en esta estructura. Para diferenciar a la población que ha experimentado una infección natural, también se pueden detectar los anticuerpos anti-nucleocápside (Anti-N) en suero. La cuantificación de los títulos de anticuerpos Anti-S permite evaluar el grado de respuesta a la vacuna, pudiendo también confirmar una infección natural, si se detecta un incremento sin haberse administrado una dosis de refuerzo y/o se observa una seroconversión de Anti-N. Del mismo modo, se puede confirmar una reinfección evaluando el incremento en los niveles de anticuerpos contra estas dos dianas. La OMS ha desarrollado un estándar internacional (código NIBSC 20/136) cuyos valores se expresan como unidad de anticuerpos de unión (BAU, por sus siglas en inglés) por mL. Se ha propuesto un nivel de anticuerpos de 143 BAU/mL como el nivel de protección adecuada contra la infección [7], aunque éste puede ser muy variable, dependiendo de las VOC circulantes y, por tanto, no debería ser empleado como único marcador para evaluar la necesidad de administrar una dosis de refuerzo.

La presencia de anticuerpos neutralizantes está relacionada con la protección frente a la infección por el virus SARS-CoV-2. Sin embargo, la circulación de VOC con un elevado índice de mutaciones en la proteína *Spike*, como Omicron BA.1 o BA.2, reduce la capacidad de protección de estos anticuerpos generados por la vacuna o por una infección previa [8]. Además de la neutralización, se ha subrayado recientemente la importancia de la acción mediada por la fracción Fc de anticuerpos no neutralizantes a la hora de proteger frente a la progresión a enfermedad severa, ya que induce la generación de macrófagos y células NK. Así mismo, este efecto protector entre los individuos vacunados o tras una infección natural no depende tanto de las VOC circulantes [9].

Tanto las vacunas como la infección natural por SARS-CoV-2 o coronavirus similares inducen la generación de células T específicas, con capacidad efectora y memoria inmunológica. La acción de las células T es esencial para reducir la gravedad de la infección y facilitar la recuperación del individuo infectado. Del mismo modo, su efecto protector puede perdurar durante años y no depende de la VOC infectante, ya que existe un efecto de protección cruzada inducido por diferentes coronavirus [10].

El análisis de células T específicas es más costoso, complejo y lento que la serología, ya que requiere la estimulación de células viables con antígenos específicos del SARS-CoV-2 y la posterior medición de las citoquinas producidas (IFN-γ, IL-2). El método de referencia consiste en separar las células mononucleares de sangre periférica (PBMC) mediante gradiente Ficoll y el posterior análisis por citometría de flujo. También se han desarrollado otras pruebas en sangre total, adecuadas para su uso en el laboratorio clínico, que proporcionan resultados válidos a la hora de evaluar la respuesta celular específica [11]. Estos métodos, llamados pruebas de liberación de interferón gamma (pruebas IGRA), comercializados por diversas empresas, permiten determinar de forma rápida si se ha producido una respuesta celular efectora tras la vacunación o infección. Para ello, se estimula la sangre del individuo con antígenos de la proteína *Spike* o de otras regiones del genoma del virus para, posteriormente, cuantificar la producción de IFN-γ.

Por tanto, los diferentes métodos disponibles permiten la detección de células T específicas, indicativo ello de un riesgo reducido de desarrollar enfermedad severa. Por otro lado, la actividad de las células T se mantiene durante más tiempo que la de los anticuerpos tras la infección o vacunación. Incluso la variante Ómicron puede ser diana de las células T generadas por las distintas vacunas, o en pacientes no vacunados que han pasado COVID-19. Recientemente, se ha publicado un estudio en el que se comunica que los individuos mantenían el 70–80% de la respuesta de las células CD4+ y CD8+ a la proteína *Spike* de la variante Ómicron, y la magnitud de las células T con reactividad cruzada fue similar para las variantes Ómicron, Beta y Delta, ya que estas células reconocen más regiones de la proteína *Spike* que los anticuerpos [12].

Para resumir, salvo que se reformulen las vacunas para mejorar la producción de anticuerpos específicos frente a las nuevas variantes, los individuos con células T específicas tras la vacunación o infección natural se beneficiarán poco de las dosis de refuerzo. De este modo, evaluar la actividad de las células T puede resultar útil a la hora de analizar individualmente la necesidad de administrar dosis de refuerzo, especialmente en la población más vulnerable, con mayor riesgo de desarrollar enfermedad grave.
